# 912. Impact of Rapid Identification and Stewardship Intervention on Coagulase-negative *Staphylococcus* Bloodstream Infection (BSI)

**DOI:** 10.1093/ofid/ofac492.757

**Published:** 2022-12-15

**Authors:** Eli S Goshorn, Alex Viehman, J Ryan Bariola, Tina Khadem, Brian Potoski, Ryan K Shields

**Affiliations:** Rutgers New Jersey Medical School, South Orange, New Jersey; University of Pittsburgh, Pittsburgh, Pennsylvania; UPMC, Pittsburgh, Pennsylvania; UPMC, Pittsburgh, Pennsylvania; University of Pittsburgh, Pittsburgh, Pennsylvania; University of Pittsburgh, Pittsburgh, Pennsylvania

## Abstract

**Background:**

Coagulase-negative staphylococci (CoNS) are commonly isolated from blood cultures (BCx). Most do not require treatment (tx), yet antibiotics are frequently initiated. Workup of CoNS BSI consumes significant resources. We aimed to demonstrate the safety and efficacy of an early algorithm-based rapid diagnostic testing (RDT) plus antimicrobial stewardship (ASP) intervention.

**Methods:**

BCx with CoNS were captured in 3 time periods to represent pre-RDT, RDT-only, and RDT+ASP. GenMark ePlex RDT was implemented for all Gram-positive BCx, identifying *S. epidermidis* and other non-aureus/lugdunensis *Staphylococcal* species. Results were called to ASP in both RDT-only and RDT+ASP periods. In the latter, a prospective algorithm was implemented to standardize ASP recommendations (**Fig 1**) for cases classified as simple, uncomplicated, and complicated BSI. The primary outcome was receipt of < 24h of antibiotic tx. Safety outcomes included rates of recurrent BSI and hospital readmission.

**Figure:**
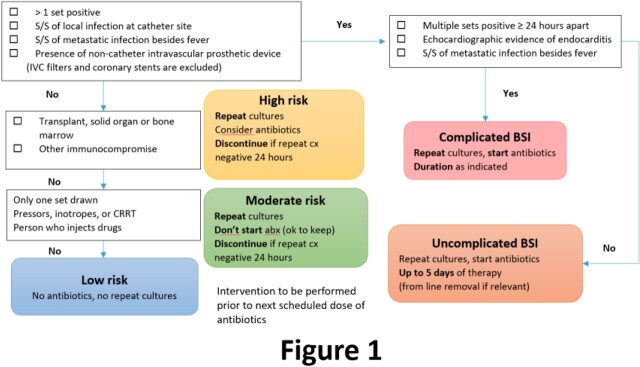

**Results:**

184 patients were included (**Fig 2**). The median age was 62, 54% were male, and the median Charlson comorbidity index was 5. 41% had a central venous catheter at time of BSI. Overall, 67%, 19%, and 12% of cases were classified as simple, uncomplicated, or complicated BSI, respectively (**Fig 3**). BSI class and patient demographics did not vary between periods. During pre- and post-RDT periods, median days of antibiotic tx did not vary significantly for patients with simple BSI (1.1 vs 1.2). In contrast, median days of tx were reduced to 0 *(P*=0.005) for simple BSI in the RDT+ASP period. Overall, 54% of patients with CoNS BSI received < 24h of tx in RDT+ASP time period compared to 34% (*P*=0.009) in the combined pre-RDT and RDT-only periods. Tx was entirely avoided in 28% of CoNS BSI cases in the RDT+ASP period compared to 16.5% of cases in other periods (*P*=0.07). 7 cases classified as simple were reclassified as uncomplicated or complicated after further work up. Rates of recurrent BSI and 30-day readmission were comparable across time periods.

**Figure:**
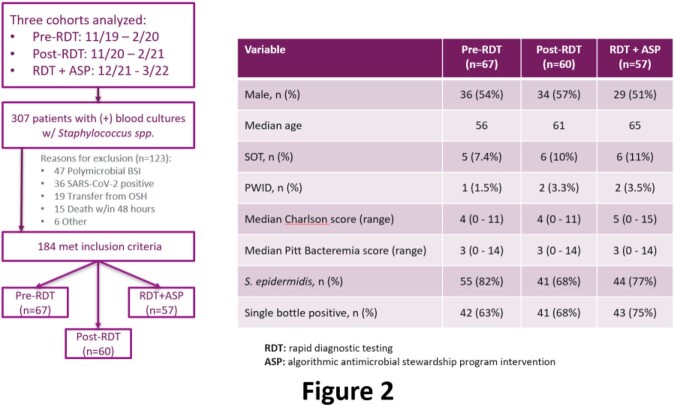

**Figure:**
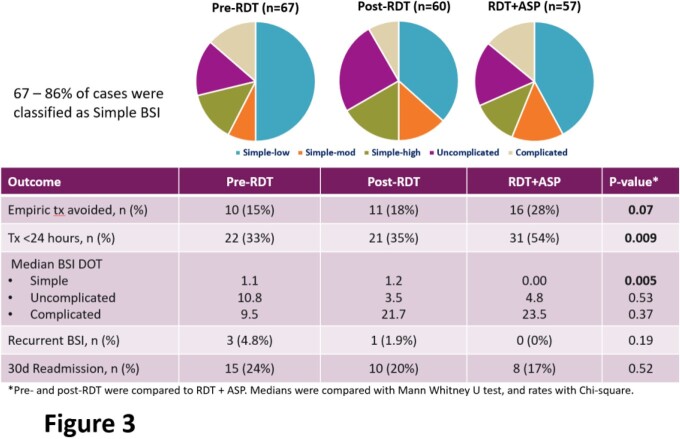

**Conclusion:**

Our algorithm-based ASP + RDT intervention reduced antibiotic tx for CoNS BSI, an effect not realized with RDT alone. These data attest to the safety and efficacy of early ASP intervention for patients with CoNS BSI identified by RDTs at the time of positive BCx.

**Disclosures:**

**J Ryan Bariola, MD**, Infectious Disease Connect: Salary support|Merck: Grant/Research Support **Tina Khadem, PharmD**, Infectious Disease Connect: Salary support|Merck: Grant/Research Support **Brian Potoski, PharmD, BCPS-AQ ID**, Merck Group: Grant/Research Support **Ryan K. Shields, PharmD, MS**, Infectious Disease Connect: Advisor/Consultant|Merck: Advisor/Consultant|Merck: Grant/Research Support|Roche: Grant/Research Support.

